# The stress-protectant molecule trehalose mediates fluconazole tolerance in *Candida glabrata*

**DOI:** 10.1128/aac.01349-24

**Published:** 2025-01-24

**Authors:** Qingjuan Zhu, Stefanie Wijnants, Regina Feil, Wouter Van Genechten, Rudy Vergauwen, Odessa Van Goethem, John E. Lunn, Mieke Van Ende, Patrick Van Dijck

**Affiliations:** 1 Department of Biology, Laboratory of Molecular Cell Biology, Institute of Botany and Microbiology, KU Leuven, Leuven, Belgium; 2 Max Planck Institute of Molecular Plant Physiology 28322 , Potsdam-Golm, Germany; 3 Leuven One Health Institute, KU Leuven 26657 , Leuven, Belgium; University of Iowa, Iowa City, Iowa, USA

**Keywords:** *Candida glabrata*, *TPS1*, *TPS2*, *NTH1*, trehalose, trehalose 6-phosphate, antifungal susceptibility, fluconazole, tolerance, ergosterol

## Abstract

The incidence of non-*albicans Candida* infections has witnessed a substantial rise in recent decades. *Candida glabrata (Nakaseomyces glabratus*), an opportunistic human fungal pathogen, is accountable for both superficial mucosal and life-threatening bloodstream infections, particularly in immunocompromised individuals. Distinguished by its remarkable resilience to environmental stressors, *C. glabrata* exhibits intrinsic tolerance to azoles and a high propensity to swiftly develop azole resistance during treatment. The molecular mechanism for the high tolerance is not fully understood. In this work, we investigated the possible role of trehalose in this tolerance. We generated mutants in the *C. glabrata TPS1*, *TPS2*, and *NTH1* genes, encoding trehalose 6-phosphate synthase (Tps1), trehalose 6-phosphate phosphatase (Tps2), and neutral trehalase (Nth1), respectively. As expected, the *tps1∆* strain cannot grow on glucose. The *tps2*∆ strain demonstrated diminished trehalose accumulation and very high levels of trehalose 6-phosphate (T6P), the biosynthetic intermediate, in comparison to the wild-type (WT) strain. Whereas these higher T6P levels did not affect growth, the lower trehalose levels clearly resulted in lower environmental stress tolerance and a lower susceptibility to fluconazole. More interestingly, the *tps2∆* strain completely lost tolerance to fluconazole, characterized by the absence of slow growth at supra-MIC concentrations of this drug. All these phenotypes are reversed in the *nth1*∆ strain, which accumulates high levels of trehalose. Our findings underscore the role of trehalose in enabling tolerance toward fluconazole in *C. glabrata*. We further show that the change in tolerance is a result of the effect that trehalose has on the sterol pattern in the cell.

## INTRODUCTION

Fungi infect billions of people every year but still remain largely under-appreciated as human pathogens (1, 2). Among candidemia cases, only five species, namely, *C. albicans, C. glabrata, C. tropicalis, C. parapsilosis,* and *C. krusei,* account for 92% of the total (3, 4). However, their distribution varies in population-based studies conducted in different geographical areas. *C. albicans* is the most encountered species, but there are significant differences in the number of cases caused by *C. glabrata* and *C. parapsilosis* (5). Studies from Northern Europe and the United States have reported a high prevalence of candidemia caused by *C. glabrata* (3). Isolates of this species are a threat to human health due to their ability to rapidly develop resistance to antifungal agents (6). Unlike other *Candida* species that are diploid and usually require alterations in both alleles to confer resistance, *C. glabrata* is a haploid organism and only a single amino acid alteration may be required to confer resistance (7). Therefore, greater effort is necessary to improve the available antifungals, as well as to find more potent and safer compounds with fungicidal action, or that prevent virulence attributes to be expressed. *C. glabrata* infections remain a clinical challenge, and there is an urgent need for new antifungal drugs with a novel mode of action to address the rising incidence of such infections (8).

**Fig 1:**
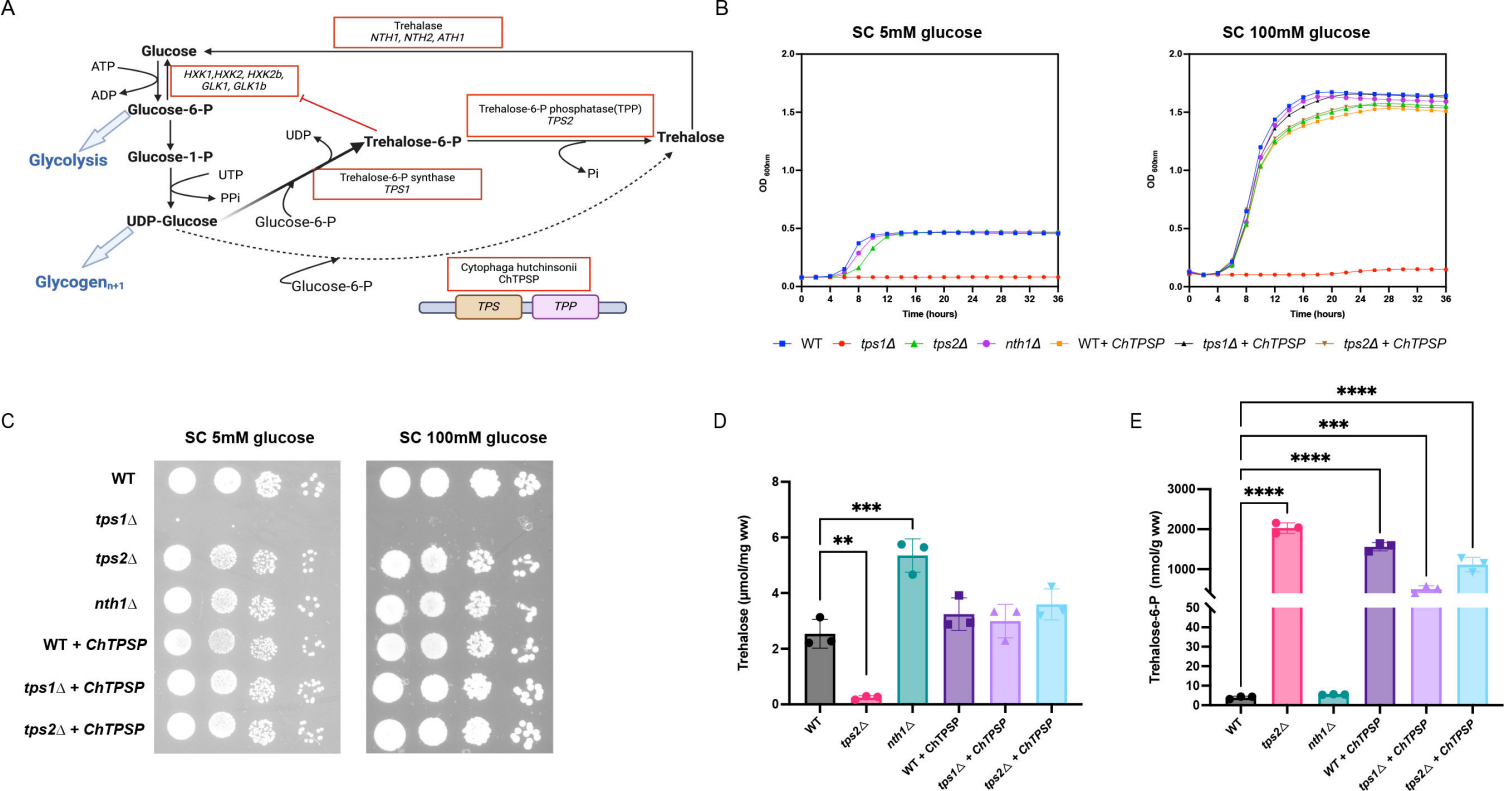
Deletion of *TPS1* affects the utilization of glucose as a carbon source. *C. glabrata* cells (WT, *tps1∆*, *tps2∆*, *nth1∆,* WT *+ ChTPSP, tps1∆ + ChTPSP,* and *tps2∆ + ChTPSP* were grown at 37°C in a liquid medium and growth was monitored using a Multiskan microplate photometer. (**A**) A schematic diagram of the pathway of trehalose metabolism in *C. glabrata*. G-6-P is glucose-6-phosphate, UDPG is uridine-5-diphosphoglucose, Trehalose-6-P is trehalose-6-phosphate. (**B**) The different strains were grown in SC medium supplemented with 5 mM glucose or 100 mM glucose. The OD_600nm_ was followed over time for 48 h. The data shown represent one biological replicate (WT, *tps1∆-*1, *tps2∆-*1, *nth1∆-*1*,* WT *+ ChTPSP*-1*, tps1∆ + ChTPSP*-1, and *tps2∆ + ChTPSP*-1), with this result being representative of all three independent mutant strains tested. (**C**) The various strains were grown on a solid SC medium supplemented with 5 mM glucose or 100 mM glucose. The plates were incubated for 48 h at 37°C before pictures were taken. The same strains as in panel B were used. (**D**) Trehalose levels of the different strains grown till exponential phase in SC plus 100 mM glucose at 37°C, 200 rpm. Each dot represents a different independent transformant. (**E**) T6P levels of the different strains growing till exponential phase in SC plus 100 mM glucose at 37°C, 200 rpm. Each dot represents a different independent transformant. Average T6P and trehalose levels with SEM are shown in two experiments using three independent transformants of each strain. Statistical analysis was conducted by two-way ANOVA with Bonferroni correction; ns, no significant, *P < 0.05; **P < 0.01; ***P < 0.001; ****P < 0.0001.

Trehalose, characterized by its chemical structure as α-D-glucopyranosyl- (1,1)-α-D-glucopyranoside, is a non-reducing disaccharide consisting of two glucose moieties. Its significance in fungal conidia survival and germination is well-documented, primarily attributed to its role as a carbon source (9). However, trehalose is also very important to provide protection against various stresses, including antifungal drugs (10, 11). Trehalose is produced through a two-step enzymatic process involving trehalose-6-phosphate synthase (Tps1) and trehalose 6-phosphate phosphatase (Tps2) (Fig. 1A). Initially, Tps1 catalyzes the conversion of uridine diphosphate (UDP)-glucose and glucose 6-phosphate into trehalose-6-phosphate (T6P) and UDP. Subsequently, Tps2 converts T6P into trehalose and phosphate (12, 13). Conversely, the breakdown of trehalose primarily occurs via hydrolysis facilitated by an α-glucosidase enzyme, which exhibits specificity toward trehalose as its exclusive substrate. This hydrolytic activity is carried out by trehalase enzymes. In *C. glabrata,* these are encoded by *ATH1*, *NTH1*, and *NTH2* and we previously showed their importance in virulence in a gut colonization model (14). The exploration of enzymes implicated in trehalose biosynthesis as potential antifungal targets is based on their absence in humans (15–17). The significant role of trehalose in virulence has been demonstrated in various pathogens, both non-fungal and fungal. In non-fungal pathogens, trehalose biosynthesis has been found to enhance the pathogenicity of *Pseudomonas aeruginosa* in plants (18). In fungal pathogens such as the opportunistic yeast *C. albicans* (19–21), the filamentous fungus *Aspergillus fumigatus (A. fumigatus)* (22, 23), the phytopathogenic fungus *Magnaporthe oryzae (M. oryzae)* (24), and the meningitis-inducing basidiomycete *Cryptococcus neoformans (C. neoformans)* (25), trehalose also plays a crucial role in promoting virulence. These studies underscore the potential of trehalose metabolism as a promising avenue for antifungal intervention, offering insights into the vulnerabilities of diverse fungal pathogens (26). Whereas *C. glabrata* is phylogenetically very close to *Saccharomyces cerevisiae* (*S. cerevisiae*), the role of trehalose metabolism in the virulence of this pathogen has not yet been investigated. *C. glabrata* expresses one *TPS1*, one *TPS2* gene, and three trehalase encoding genes (14).

In this work, we generated deletion strains in the trehalose biosynthesis genes *TPS1* and *TPS2*, and we also generated a new *NTH1* deletion strain using the phototrophic ATCC2001 strain as the background, which differs from the Van Ende study that used a background strain with multiple auxotrophies (ATCC2001 *his3∆ trp1∆ leu2∆*) (14). We also generated strains where we expressed the *Cytophaga hutchinsonii* (*C. hutchinsonii*) *TPSP* gene (*ChTPSP*), a natural TPS–TPP bifunctional enzyme present in this bacterial species (27), into the WT, *tps1*Δ, and *tps2*Δ strains. This was undertaken to elucidate whether T6P or trehalose serves as the primary determinant for stress resistance or fluconazole tolerance. Our comprehensive analysis has revealed that the diminished intracellular trehalose levels, more than elevated T6P levels observed in the *tps2*Δ strain, correlate with increased susceptibility to thermal, oxidative, and osmotic stresses and a complete absence of tolerance to fluconazole. Conversely, the *nth1*Δ strain, which accumulates higher trehalose levels, showed opposite phenotypes with an increase in tolerance toward fluconazole. Of particular interest is the observation that the difference in drug tolerance between the *tps2Δ* and *nth1Δ* strains may be attributed to the role that trehalose plays in membrane stabilization. This stabilization likely affects drug efflux activity, which, in turn, influences the sterol composition in these mutant strains.

## RESULTS

### The *C. glabrata TPS1* gene is required for growth on glucose as a carbon source

The Tps1 enzyme of *S. cerevisiae* is required for growth on glucose as deletion of this gene results in uncontrolled influx of glucose into glycolysis, resulting in accumulation of sugar phosphates and concomitant depletion of ATP leading to apoptosis (28, 29). In *C. albicans*, deletion of *TPS1* results in a growth defect on glucose, but only at temperatures above 39°C (30). To determine whether the Tps1 enzyme of *C. glabrata* is also involved in growth on glucose, we tested the different strains on SC medium supplemented with physiological glucose levels (5 mM) as well as with high glucose levels (100 mM). Our results show that the *TPS1* gene is essential for utilizing glucose as a carbon source since the *tps1∆* strain cannot grow in either liquid culture or on solid medium with glucose as the carbon source (Fig. 1B and C). Similar results were obtained in the presence of trehalose or fructose as the carbon source (Fig. S1). *C. glabrata* has a problem in using galactose as a carbon source, and this was clearly visible in our experiments, where all strains showed a slow growth phenotype on galactose-containing media (Fig. S1). Deletion of the *TPS2* or *NTH1* genes had no obvious effects on growth on any of the carbon sources in liquid or on solid media (Fig. S1 and S2). We show that the *tps2∆* strain accumulates high levels of T6P and low levels of trehalose and that under optimal growth conditions, the WT strain will have low T6P and low trehalose levels.

### Expression of the bifunctional *C. hutchinsonii TPSP* gene still resulted in high levels of T6P upon expression in the *tps1∆* strain

To understand the role of Tps1 or Tps2, we needed to determine the levels of T6P and trehalose as these metabolite levels may contribute to the phenotype of the mutants. As the *tps1∆* strain does not grow on glucose, regular physiologically relevant growth conditions are incompatible with this strain. Therefore, we expressed the bifunctional T6P synthase/phosphatase enzyme (TPSP) of *C. hutchinsonii* (27) in the *tps1∆* strain with the aim to obtain a strain that has little T6P, as we expect efficient conversion of T6P into trehalose in this enzyme, but high levels of trehalose. Expression of the *ChTPSP* gene in the *tps1*∆ strain restored growth on glucose, which was expected as the *ChTPSP* enzyme possesses both TPS and TPP enzymatic activities (Fig. 1B and C) (27). However, expression of *ChTPSP* in the *tps1∆* mutant resulted in only the same level of trehalose as observed for the WT and a *tps2∆* strain expressing the bifunctional enzyme (Fig. 1D). Unexpectedly, expression of the bifunctional enzyme in the *tps1∆* strain resulted in high levels of T6P (Fig. 1E). These levels were still much lower than those obtained for the *tps2∆* strain. As we previously observed in *C. albicans*, deletion of *TPS2* still results in some accumulation of trehalose. Deletion of *NTH1* resulted in increased trehalose levels as expected, and it had no effect on T6P levels. Based on these results, the remainder of the manuscript will focus on the WT, the *tps2∆* strain, and the *nth1∆* strain as these strains have normal, high, or low trehalose levels.

### Trehalose levels are important for heat, oxidative, membrane and salt stress tolerance

**Fig 2:**
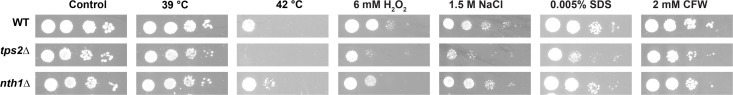
Trehalose provides tolerance against heat stress and is required for oxidative, membrane, and salt stress tolerance. The different strains were grown overnight in SC medium with 100 mM glucose at 37°C. After three washing steps, 10-fold dilutions of cells were spotted on SC plus 100 mM glucose agar plates challenged with high temperature stress (at 39°C or 42°C), or with oxidative (H_2_O_2_) or salt (NaCl) stress or membrane (SDS) or cell wall (CFW) stress and imaged after 48 h of growth. The data shown represent one biological replicate (WT*, tps2∆-*1, and *nth1∆-*1), with this result being representative of all three independent mutant strains tested.

Inside the human body, fungal pathogens are continuously exposed to different types of environmental stresses (31). As trehalose is a disaccharide important for stress resistance (32), we assessed the growth phenotype of the deletion strains upon different stress treatments: oxidative stress (H_2_O_2_), salt stress (NaCl), membrane stress (SDS, Sodium Dodecyl Sulfate), cell wall stress (CFW, Calcofluor-white) and heat stress (39°C and 42°C). Heat stress of 42°C exceeds that of a human fever, but was chosen in accordance with previous studies, where this temperature was often used as a test condition for heat stress (33). The WT could clearly not grow at this temperature, but the *nth1∆* strain still grows, suggesting that the high amount of trehalose in these cells protects the cells against this stress (Fig. 2). An opposite trend was observed under oxidative stress conditions, but the difference between the WT and the *nth1∆* strain was minor. We did not observe any differences between the WT and the *nth1∆* strain under osmotic stress conditions (Fig. 2). The *tps2∆* strain shows lower tolerance at 42°C compared to the WT strain and lower tolerance toward oxidative, membrane, and salt stress conditions (Fig. 2). Regarding cell wall stress, we did not observe any significant differences in growth between the WT strain and the mutant strains.

### Trehalose accumulation increases resistance and tolerance to fluconazole in *C. glabrata*

**Fig 3:**
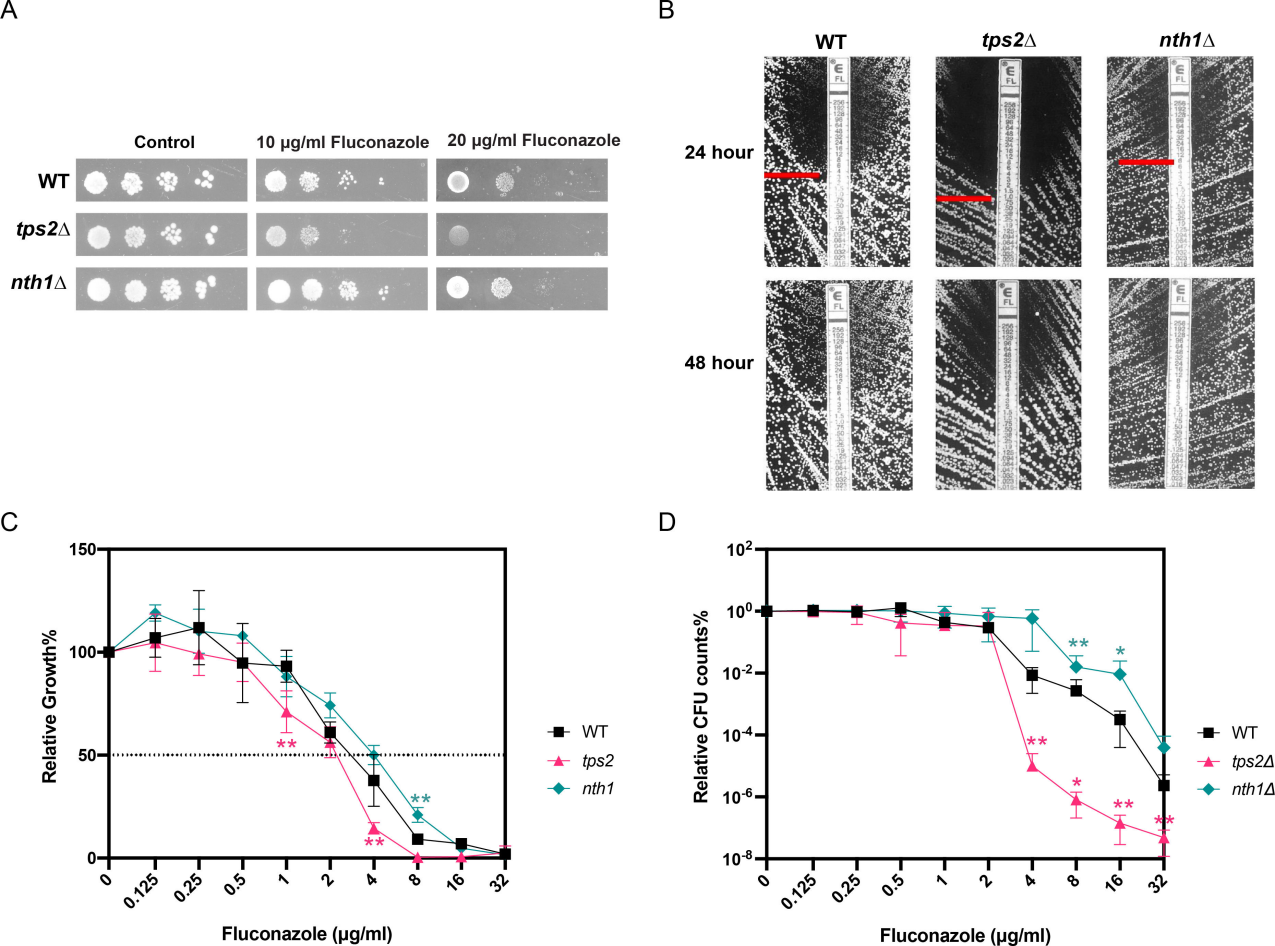
Trehalose plays an important role in tolerance against fluconazole. (**A**) Serial dilutions of the WT, *tps2∆,* and *nth1∆* strains spotted on SC glucose plates. Pictures were taken after 48 h of incubation at 37°C. (**B**) Etest analysis at 37°C (24 and 48 h) showing a “halo” in regions of the medium with high concentrations of fluconazole where the cells are unable to grow. The data shown represent one biological replicate (WT, *tps2∆-*1, and *nth1∆-*1). (**C**) Growth profiles of mutant strains relative to the WT strain in a broth dilution assay (BDA) in the presence of different concentrations of fluconazole. The experiment was conducted with three biological and three technical repeats, and representative results are shown. (**D**) Tolerance assay. Data reflect the relative percentage of colony-forming units from the WT and mutant strains after 48 h of incubation at 37°C. Statistical analysis was conducted by two-way ANOVA with Bonferroni correction; *P < 0.05; **P < 0.01. The experiment was conducted with three biological and three technical repeats, and representative results are shown.

Trehalose metabolism enzymes are interesting antifungal drug targets, and several groups are trying to find drugs that target these enzymes, but as far as we know, no compound targeting these enzymes is in any clinical trial (17, 34, 35). Such inhibitors could also work synergistically with known antifungals as it was already shown in *C. albicans* that trehalose-deficient mutants are sensitive to amphotericin B and micafungin (21). In *C. glabrata*, no significant differences in sensitivity toward amphotericin B and caspofungin were observed between the WT, *tps2∆,* or *nth1∆* strains, as assessed by Epsilometer test (Etest) analysis (Fig. S3A) and broth dilution assays (BDA) (Fig. S3B). Unlike other typical *Candida* species, *C. glabrata* inherently exhibits reduced susceptibility to azole drugs, particularly fluconazole. To determine a possible role for trehalose metabolism in this fluconazole tolerance or resistance, we tested the growth of WT, *tps2*Δ, and *nth1*Δ strains in the presence and absence of fluconazole (Fig. 3A). Deletion of *TPS2* resulted in strains that are sensitive to fluconazole treatment, while the absence of the *NTH1* gene resulted in improved growth under fluconazole stress conditions.

The minimum inhibitory concentration of fluconazole (MIC_flu_) for these strains was determined through Etest (Fig. 3B) and BDAs (Fig. 3C and D). From the Etest results, it can be inferred that the absence of the *NTH1* gene results in a decreased susceptibility to fluconazole, while deletion of the *TPS2* gene renders the cells more susceptible to fluconazole. The MIC_50_ values we obtain here are 8 µg/mL for the WT, 2 µg/mL for the *tps2∆* strain, and 12 µg/mL for the *nth1∆* strain. These data differ from the results obtained using the BDA where the difference between the strains is less clear with MIC_50_ of 4 µg/mL for WT, 2–4 µg/mL for *tps2∆,* and 4–8 µg/mL for the *nth1∆* strain. The BDA method not only allows for the determination of the MIC50 value but also provides insight into tolerance by measuring growth over 48 h at supra-MIC levels of fluconazole. Figure 3D shows that there is a strong difference in colony-forming unit (CFU) counts at higher concentrations of fluconazole between WT and mutant strains. The *tps2∆* strain exhibits reduced tolerance, particularly at fluconazole concentrations above the MIC₅₀, resulting in a reduced survival rate of the *tps2∆* compared to the WT. In contrast, the *nth1∆* strain demonstrates increased tolerance, as *nth1∆* cells show a significantly higher survival rate above MIC₅₀ concentrations compared to the WT strain. Furthermore, we observed that introducing the *ChTPSP* gene into the *tps2∆* strain restored fluconazole resistance to levels comparable to those of the WT strain (Fig. S4).

**Fig 4:**
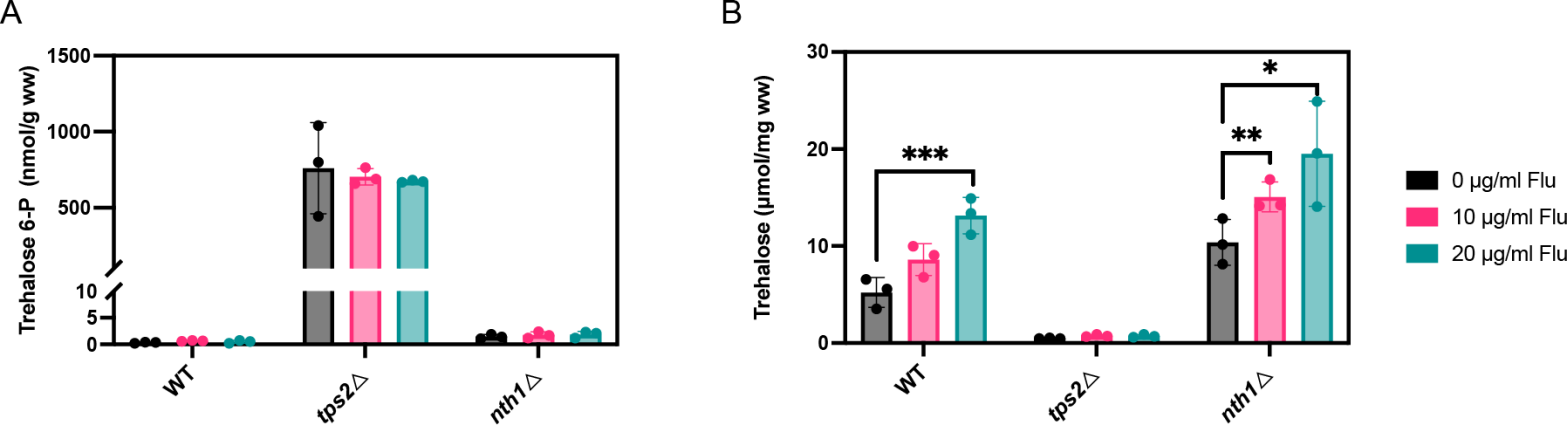
Intracellular T6P and trehalose levels in response to fluconazole stress. (**A**) T6P levels after fluconazole treatment. The cultures were divided in three and incubated further at 37°C with or without fluconazole for 60 min. (**B**) Trehalose levels in the presence and absence of fluconazole. The cultures were divided into three and incubated further at 37°C with or without fluconazole for 60 min (ww, wet weight). Average T6P and trehalose levels with SEM are shown for three independent transformants of each strain. Each dot represents a different independent transformant. Statistical analysis was conducted by two-way ANOVA with Bonferroni correction; ns, no significant, *P < 0.05; **P < 0.01; ***P < 0.001.

Additionally, we quantified the intracellular levels of trehalose and T6P to elucidate whether trehalose or T6P influences the resistance and tolerance to fluconazole in *C. glabrata*. In the analyzed strains, following a 60 min exposure to fluconazole, both the WT and *nth1*Δ strains accumulated trehalose in response to the antifungal challenge, with a significantly increased accumulation observed in the *nth1*Δ strain especially upon fluconazole treatment (Fig. 4A). However, there was no change in T6P levels observed in any of the three strains following fluconazole treatment (Fig. 4B). These findings indicate that trehalose levels are significantly upregulated in response to fluconazole stress. This upregulation may play an important role in resistance and tolerance to the drug.

### Impact of trehalose accumulation on residual ergosterol levels and *ERG11* gene expression

**Fig 5:**
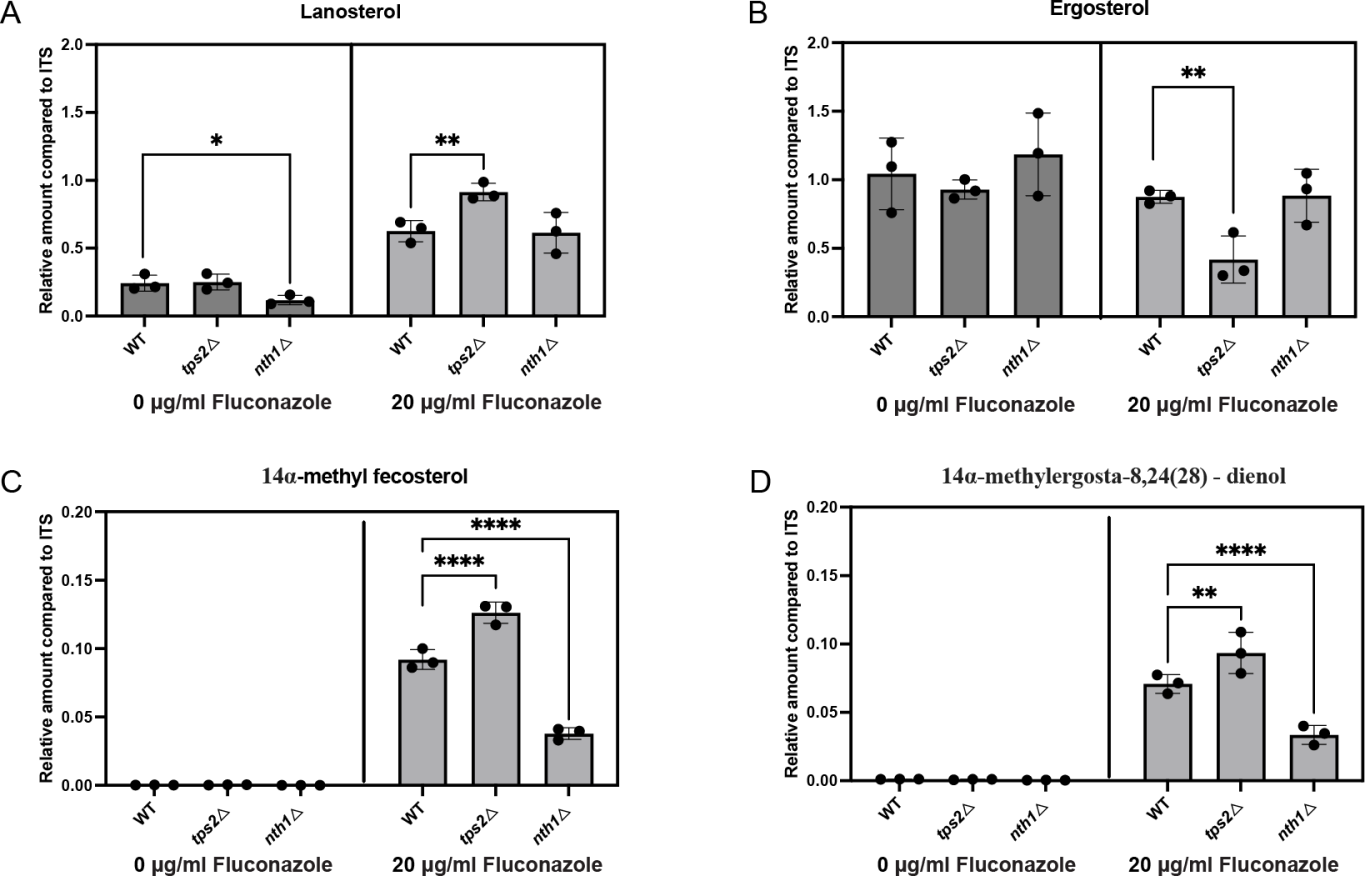
Trehalose metabolism affects the sterol content in *C. glabrata*. (**A–D**) Strains were grown in SC glucose medium for 24 h in the absence or presence of fluconazole. Sterol levels were determined by GC-MS and are displayed for lanosterol, ergosterol, 14α-methyl fecosterol, and 14α-methylergosta-8,24(28)-dienol. The values are calculated relative to the internal standard (ITS; cholestane). Data of the relative amount of sterol compared to ITS, with SEM, are shown from two experiments using three independent transformants of each strain. Each dot represents a different independent transformant. Statistical analysis was conducted by two-way ANOVA with Bonferroni correction; *P < 0.05; **P < 0.01; ***P < 0.001; ****P < 0.0001.

Fluconazole targets Erg11, leading to an accumulation of lanosterol and a decrease of ergosterol levels in the presence of the drug (36). This presence also prompts the conversion of lanosterol to 14-methylfecosterol and ultimately to the toxic compound 14α-methylergosta-8,24(28)-dienol, which constitutes a crucial aspect of the drug’s mode of action (37, 38). To explore the potential involvement of ergosterol in mediating the impact of trehalose metabolism-related enzymes on fluconazole susceptibility, we assessed *ERG11* gene expression levels and ergosterol levels in the mutant strains. In the absence of fluconazole, only a slight difference was observed between the WT and mutant strains regarding lanosterol and ergosterol levels (Fig. 5). Under conditions without fluconazole, the deletion of *NTH1* showed a trend toward increased ergosterol levels, while the *tps2Δ* strain appeared to have reduced ergosterol levels. However, these differences were not statistically significant (*P* > 0.05). Furthermore, there was no production of 14α-methyl fecosterol and toxic sterol [14α-methylergosta-8,24(28)-dienol] observed. Upon fluconazole treatment, no significant differences in ergosterol levels were observed between the WT and the *nth1Δ* strain. However, the *tps2Δ* strain demonstrated a marked decrease in ergosterol levels compared to the WT strain, suggesting a distinct alteration in ergosterol biosynthesis upon fluconazole exposure in the absence of Tps2. Remarkably, we observed that the absence of the *NTH1* gene resulted in less 14α-methyl fecosterol, and toxic sterol formation compared to the WT strain. Conversely, the absence of *TPS2* led to a significant increase in the accumulation of 14α-methyl fecosterol and the formation of toxic sterols. In summary, it appears that reduced trehalose levels correlate with decreased ergosterol synthesis and higher levels of toxic sterols, which may be the reason for the reduced tolerance.

**Fig 6:**
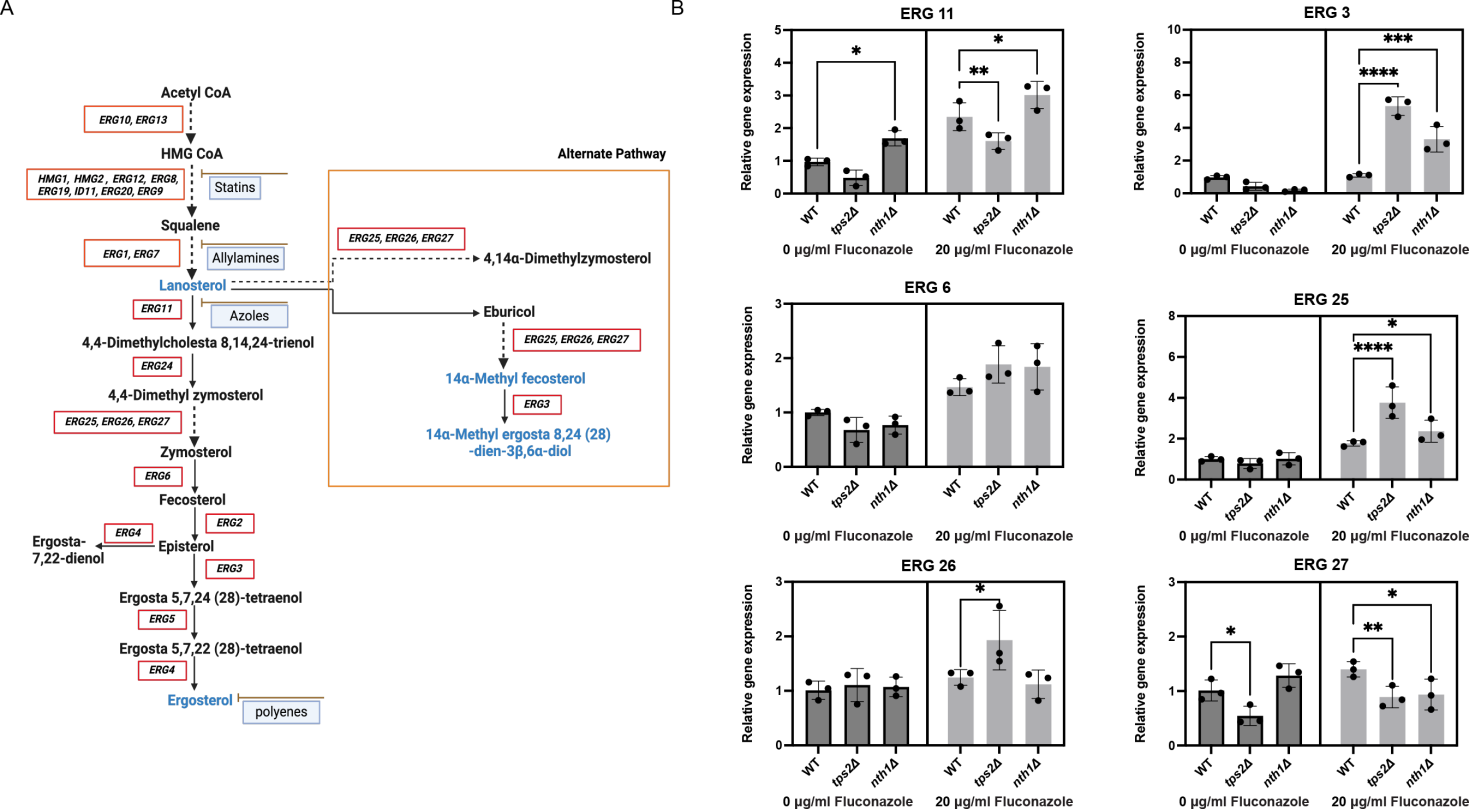
Quantitative RT-PCR determination of the *ERG* gene expression in response to fluconazole stress. (**A**) Representation of the ergosterol biosynthesis pathway in *C. glabrata*. CoA, coenzyme. (**B**) Exponentially growing WT, *tps2Δ,* and *nth1Δ* strains, in SC glucose medium at 37°C, were incubated with 20 µg/mL fluconazole for 60 min. Gene expression was analyzed using quantitative reverse transcriptase PCR. Relative gene expression level with SEM are shown from two experiments, each with three independent transformants. The values were calculated relative to the average of the values from the WT untreated samples. Each dot represents a different independent transformant. Statistical one-way analysis of variance with Bonferroni correction was done on the log transformed values (*P ≤ 0.05, **P ≤ 0.01).

As Erg11 is the target of fluconazole and catalyzes the step in the sterol biosynthesis pathway where the flux toward ergosterol or to toxic sterols is defined (Fig. 6A) (39), it seems valid to hypothesize that Erg11 might be the enzyme linking trehalose, or its metabolic enzymes, to ergosterol biosynthesis. We investigated the gene expression levels of genes involved in the ergosterol biosynthesis pathway (*ERG11, ERG3, ERG6, ERG25, ERG26,* and *ERG27*) in the presence and absence of fluconazole. In this way, we could determine whether differences in sterol intermediates are due to different levels of gene expression. Under both conditions, deletion of *NTH1* results in a significant increase in *ERG11* expression, compared to the WT strain (Fig. 6B). Deletion of *TPS2* results in a lower expression of *ERG11* (Fig. 6B). Furthermore, we found that deletion of *TPS2* significantly upregulates the expression of *ERG3, ERG25,* and *ERG26* under fluconazole-treated conditions (Fig. 6B) compared to the WT strain. Additionally, an increase in the expression of *ERG6* and *ERG27* was observed although these changes were not statistically significant. This difference in gene expression may be the reason why the *tps2∆* strain exhibited increased levels of toxic sterols and, therefore, contribute to the reduced fluconazole stress tolerance of this mutant.

### Absence of trehalose results in lower drug efflux pump gene expression impairs antifungal resistance by regulating efflux pump genes as determined by a rhodamine 6G efflux assay

**Fig 7:**
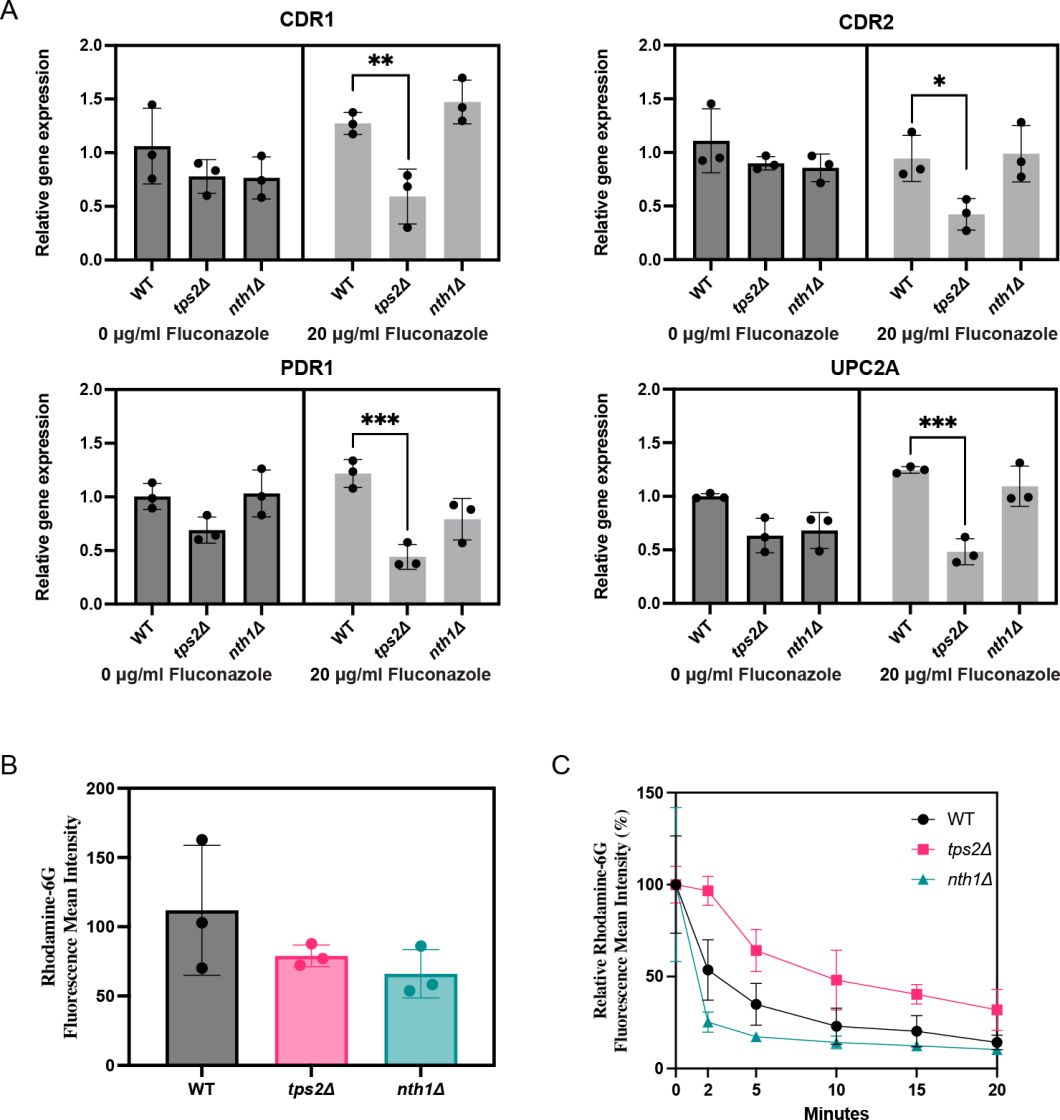
Trehalose Impairs fluconazole resistance by regulating efflux pump genes and affecting rhodamine 6G efflux activity. (**A**) Exponentially growing WT, *tps2Δ,* and *nth1Δ* strains, in SC glucose medium at 37°C, were incubated with 20 µg/mL fluconazole for 60 min. Gene expression was analyzed using quantitative reverse transcriptase PCR. Relative gene expression level with SEM is shown from two experiments, each with three independent transformants. The values were calculated relative to the average of the values from the WT untreated samples. Each dot represents a different independent transformant. Statistical one-way analysis of variance with Bonferroni correction was done on the log transformed values (*P ≤ 0.05, **P ≤ 0.01). (**B**) Comparison of intracellular rhodamine 6G accumulation between strains, with results represented as the geometric mean of fluorescence intensity. Data with SEM are presented as the average of three biological replicates. Each dot represents a different independent transformant. No significant difference was observed in R6G accumulation between the mutant strains and the WT strain (*P* = 0.731 for WT vs *tps2Δ*, *P* = 0.106 for WT vs *nth1Δ*). (**C**) Analysis of efﬂux pump activity by comparison of R6G efﬂux, in relative ﬂuorescence units (RFU), after the addition of glucose. Data are presented as the average of three biological replicates with SEM. Statistical significance was assessed using nonlinear regression (curve fitting) analysis, followed by an extra SUM-of-Squares *F* test for the rate constant (*****K*****), where *P* ≤ 0.05 is considered significant (*P* = 0.0151 for WT vs *tps2Δ*, *P* = 0.0557 for WT vs *nth1Δ*).

In *C. glabrata*, resistance to antifungal drugs often involves overexpression of multidrug transporter genes such as *CDR1* and *CDR2*, and the transcriptional regulators *PDR1* and *UPC2A*, which together contribute to complex drug resistance mechanisms. To understand the role of trehalose in the regulation of the expression levels of these genes, we performed a gene expression analysis in the presence and absence of fluconazole treatment. Deletion of *TPS2* resulted in a significant downregulation of *CDR1*, *CDR2*, *PDR1*, and *UPC2A* in response to fluconazole exposure (Fig. 7A). No notable changes in gene expression were observed in the *NTH1* deletion strain under these conditions.

To evaluate the functional impact of this reduced gene expression, we determined Rhodamine 6G (R6G) efflux. This fluorescent dye is a substrate of the Cdr1 and Cdr2 transporters. Flow cytometry analysis revealed that both the *tps2∆* and *nth1Δ* strains exhibited lower intracellular accumulation of R6G compared to the WT strain, but the difference was not statistically significant (Fig. 7B). Following glucose induction to activate the drug efflux pumps, the *nth1∆* strain had a slightly faster efflux of R6G, whereas the *tps2Δ* strain displayed a significant reduced R6G efflux activity compared to the WT strain (Fig. 7C). These results suggest that absence of trehalose in the *tps2∆* strain has a negative effect on drug efflux activity.

## DISCUSSION

In this study, mutants lacking the *TPS1*, *TPS2*, and *NTH1* genes in *C. glabrata* were generated and functionally characterized. Our findings underscored the essential role of the *TPS1* gene in glucose utilization as a carbon source, as evidenced by the complete inhibition of growth in the *tps1*Δ strain at 37°C. Similar to observations in *S. cerevisiae* (40), severe ATP depletion and hexose phosphate accumulation were observed in the *tps1*Δ strain upon glucose addition. This effect is attributed to the loss of hexokinase inhibition by T6P, leading to increased glycolytic flux, activation of the protein kinase A pathway and apoptosis (29, 41, 42). Whereas this phenotype is similar in *S. cerevisiae* and in *C. glabrata*, this is different from what was described in *C. albicans*. Deletion of *TPS1* in *C. albicans* results in a strain that can still grow on glucose at 37°C but shows growth defects on glucose at higher temperatures (30). Deletion of the *TPS2* gene results in a strain that accumulates very high levels of T6P under exponential growth conditions. Despite the absence of Tps2, there is still some accumulation of trehalose, a phenotype that was also observed in *C. albicans* and in *S. cerevisiae tps2∆* mutants, suggesting that some non-specific phosphatases are active under these conditions (43). A clear difference between *S. cerevisiae* and *C. glabrata* is the fact that under exponential growth conditions on glucose containing medium, *S. cerevisiae* does not accumulate trehalose, whereas we observe a clear accumulation in *C. glabrata* (44) (Fig. 1D). Based on the very high T6P levels in the *tps2∆* strain, we assume that the Tps1 enzyme is very active, even under exponential growth conditions in the absence of stress. Deletion of *NTH1* did not result in very high accumulation of trehalose, suggesting that this enzyme is not very active in exponentially growing cells in *C. glabrata*. Deletion of *NTH1* in *S. cerevisiae* increases trehalose levels by 50%–80% (45, 46). A clear observation that distinguishes *C. glabrata* from *S. cerevisiae* is that the human fungal pathogen always accumulates trehalose, even under non-stress conditions. Another distinction is that *C. glabrata* can accumulate very high levels of T6P without showing any growth defect.

In order to have strains that would have high trehalose levels but no accumulation of T6P, we introduced a bifunctional enzyme from *C. hutchinsonii* that was previously identified and characterized in the lab (27). Our hypothesis was that if we express the encoding gene into a *tps1∆* strain, that strain would grow on glucose and would produce high levels of trehalose and no T6P as this should be optimally channeled inside the enzyme. However, we only observed similar trehalose levels as those of the WT strain, but more importantly, there is still very high T6P accumulation (Fig. 1E). Therefore, we did not analyze the strains expressing this bifunctional enzyme any further.

The accumulation of trehalose is crucial for protecting cells against various stress conditions. Under heat stress, trehalose stabilizes proteins and membranes, preventing denaturation and ensuring cellular integrity (47). During oxidative stress, it functions as an antioxidant, scavenging reactive oxygen species (ROS) and safeguarding cellular components from damage (48, 49). In high-salt environments, trehalose acts as an osmo-protectant, helping to maintain cellular turgor and preventing dehydration (50). In our study, the growth of the *tps2Δ* strain was impaired when exposed to high temperature (42°C), oxidative stress (6 mM H_2_O_2_), salt stress (1.5 M NaCl), and membrane stress (0.005% SDS). However, there was no significant effect observed on growth under cell wall stress (2 mM CFW) (Fig. 2). Deletion of the *TPS2* gene results in a significant decrease in trehalose levels accompanied by a notable increase in T6P levels (Fig. 1D and E). The hyperaccumulation of phosphorylated intermediates such as T6P sequesters a substantial portion of the cell’s available phosphate, which can impact cellular metabolism. In consequence, oxidative phosphorylation is limited by the low availability of orthophosphate, leading to low ATP levels and cell death (22, 29, 51). This indicates that the high levels of T6P in the *tps2∆* strain under these stress conditions can result in the growth defect. In contrast, the *NTH1* deletion strain displayed increased tolerance to high temperatures (42°C). However, under salt and oxidative stress conditions, no significant changes in the growth of *nth1Δ* strain were observed compared to the WT strain. This suggests that trehalose accumulation is essential for *C. glabrata* thermotolerance and not for salt or oxidative stress tolerance.

*C. glabrata* is inherently more tolerant to fluconazole and other azoles, compared to other fungal pathogens (52). The underlying molecular mechanisms are not fully understood (53). As fluconazole causes stress in *C. glabrata* by disrupting ergosterol synthesis, resulting in membrane instability, oxidative stress, and the activation of multiple stress response pathways, we investigated the role of trehalose metabolism in fluconazole tolerance. Our results clearly show that strains with enhanced trehalose accumulation (*nth1*Δ) showed increased resistance and tolerance to fluconazole, whereas strains deficient in trehalose (*tps2*Δ) displayed increased susceptibility to the drug (Fig. 3). As T6P levels do not differ greatly between the strains upon fluconazole treatment (Fig. 4), we concluded that it is the increase in trehalose levels that enhances cellular tolerance to fluconazole, as also evidenced by the *nth1*Δ strain, which consistently maintains the same T6P levels as the WT strain. Introduction of the *ChTPSP* gene into the *tps2Δ* strain restored fluconazole sensitivity to WT strain levels, supporting the notion that trehalose influences fluconazole resistance. As trehalose is known to protect membrane structures under stress conditions (54, 55), our results may indicate that the indirect effect of fluconazole on membrane structure may be tolerated in *C. glabrata* because of trehalose accumulation. It is possible that trehalose acts as a stress protectant by stabilizing proteins and cellular membranes, preserving their structure and function under stress conditions induced by fluconazole. This stabilization helps mitigate the drug’s disruption of ergosterol biosynthesis (Fig. 5), which is crucial for maintaining fungal membrane integrity. Furthermore, its accumulation often signals the activation of stress-responsive pathways, such as the high-osmolarity glycerol (HOG) pathway, which upregulates genes associated with antifungal resistance (56–58). Further studies are necessary to clarify the specific role of trehalose and its associated pathways in modulating fluconazole sensitivity.

The increased sensitivity of the *tps2Δ* strain to fluconazole may stem from reduced trehalose levels or the accumulation of T6P. We propose that low trehalose levels increase sensitivity to fluconazole by impairing stress tolerance and membrane stability, both of which are critical defenses against the drug’s damaging effects. This reduction in cellular resilience likely makes cells more vulnerable to the metabolic and structural stress induced by fluconazole. However, it is important to acknowledge that T6P accumulation may play a significant role in this increased sensitivity as well. In some fungi, T6P is known to inhibit hexokinase (22, 59, 60), the enzyme catalyzing the initial step of glycolysis, which converts glucose to glucose-6-phosphate. When T6P accumulates, it may suppress hexokinase activity in *C. glabrata*, thereby reducing glycolytic flux. This inhibition could lead to a diminished capacity for ATP and NADPH production, both of which are essential for energy generation and biosynthetic reactions. Furthermore, the decrease in glycolytic intermediates, such as acetyl-CoA, may disrupt ergosterol biosynthesis, a critical process for maintaining fungal cell membrane integrity. Ergosterol synthesis relies on glycolytic intermediates, and disruptions in metabolic flux likely impair its production, compromising cellular function and membrane stability. Additionally, the reduced availability of ATP and NADPH further impairs the ergosterol biosynthetic pathway (61), exacerbating the vulnerability of *tps2Δ* cells to fluconazole, which specifically targets this pathway. This proposed mechanism warrants further experimental investigation to confirm the role of T6P accumulation and its downstream effects on glycolysis, energy metabolism, and ergosterol biosynthesis.

The primary target of fluconazole is Erg11, an enzyme crucial in the biosynthesis of ergosterol (62, 63). Our study indicates a significant decrease in ergosterol levels in the trehalose synthesis-deficient strain (*tps2Δ*) upon exposure to fluconazole. In contrast, the trehalose-accumulating strain (*nth1Δ*) demonstrated an ability to produce higher ergosterol levels although this increase was not statistically significant when compared to the WT strain. This suggests a protective mechanism whereby trehalose accumulation enables cells to maintain higher ergosterol levels upon fluconazole treatment. Upon detailed examination of sterol profiles in our strains, we observed that the absence of the *NTH1* gene diminishes the production of potentially toxic sterols, notably 14α-methylergosta-8,24(28)-dienol (Fig. 5C and D) (37, 38). This reduced accumulation of toxic sterols, coupled with enhanced ergosterol production under fluconazole exposure, underscores how higher trehalose levels in the *nth1∆* strain confer resistance to the drug. *ERG11* plays a pivotal role at the interface of pathways leading to either ergosterol production or toxic sterol accumulation, while *ERG3, ERG6, ERG25*, *ERG26,* and *ERG27* are responsible for both ergosterol and toxic sterol synthesis (Fig. 6A) (64). Tps2 is essential for normal levels of toxic sterols as our results show an upregulation in the expression of genes involved in their production and consequently increased levels of toxic sterols. This suggests that under fluconazole stress, trehalose accumulation protects cells by promoting the flux of the ergosterol pathway and inhibiting the production of toxic sterols. Conversely, the inhibition of Erg11 by fluconazole and the absence of trehalose result in increased levels of toxic sterols. These variations in *ERG11* expression indicate that trehalose influences tolerance by altering *ERG11* expression levels. This modification subsequently affects the metabolic flux toward either ergosterol or toxic sterols. Increased ergosterol production is associated with enhanced tolerance. This hypothesis is strengthened by our results obtained with the *nth1* mutant. Since this mutant has elevated levels of trehalose compared to the WT strain, no increase of toxic sterols is observed.

Additionally, trehalose appears to regulate the expression of drug efflux transporter genes. In *C. glabrata*, resistance to azoles is largely mediated by the overexpression of ABC transporter genes, such as *CDR1,* regulated by transcription factors Pdr1 and Upc2A (65, 66). Our findings suggest that trehalose biosynthesis plays a significant role in drug resistance by modulating the expression of efflux pump genes. Deletion of *TPS2*, resulting in low trehalose levels, leads to significant downregulation of *CDR1*, *CDR2*, *PDR1*, and *UPC2A* upon fluconazole exposure (Fig. 7A). Trehalose is essential for maintaining cellular energy homeostasis and stress responses, and its deficiency disrupts glucose sensing and metabolic regulation (67). This imbalance triggers a broader cellular response, including the downregulation of *PDR1*, a transcription factor that regulates the expression of efflux pump genes such as *CDR1* and *CDR2*. Consequently, reduced *PDR1* levels impair the induction of *CDR1* and *CDR2*, which are vital for the cell’s ability to expel antifungal drugs. Additionally, the potential role of T6P in the observed differential gene expression cannot be excluded, as this metabolite accumulates to elevated levels in the *tps2Δ* strain. Excessive T6P may interfere with proper glucose sensing and utilization, leading to further metabolic imbalances that contribute to the downregulation of *PDR1* expression. This reduction in *PDR1* levels may hinder the activation of efflux pump genes regulated by Pdr1, thereby compromising the cell’s ability to expel antifungal drugs and ultimately decreasing drug resistance. Conversely, the *nth1Δ* strain maintained normal expression levels of these genes, likely because trehalose accumulation provides sufficient protection against the drug-induced stress (Fig. 7A). The enhanced ability to manage stress ensures that the necessary transcription factors remain active, maintaining normal gene expression levels despite the fluconazole challenge.

The R6G efflux assays further support the role of trehalose in drug resistance. The *tps2*Δ strain exhibited significantly reduced R6G efflux activity following glucose induction, indicating that trehalose biosynthesis plays a key role in regulating efflux pump activity under glucose-replete conditions (Fig. 7B and C). These findings suggest that trehalose accumulation may act as a metabolic sensor, coordinating efflux pump activity with the cell’s energy status and environmental conditions.

In summary, our findings provide compelling evidence that trehalose levels play a critical role in modulating fluconazole resistance in *C. glabrata*. Specifically, trehalose influences drug efflux pump activity by regulating the expression of drug resistance genes, which subsequently modulates the expression of ergosterol biosynthesis-related genes and, in turn, affects sterol levels. Specifically, deletion of the *TPS2* gene, which results in lower trehalose levels, is associated with increased toxic sterol levels and decreased ergosterol levels (Fig. 5). The deletion of the *TPS2* gene, which leads to reduced trehalose levels, was associated with increased levels of toxic sterols and decreased levels of ergosterol. This phenomenon may be attributed to the lower drug excretion activity observed in the *TPS2* deletion strain (Fig. 7), resulting in higher intracellular drug concentrations that inhibit the expression of the *ERG11* gene and promote the accumulation of toxic sterols. Whereas in most species it is established that Tps2 is very important (but not completely essential) for trehalose biosynthesis, in *A. fumigatus,* it was shown that trehalose phosphorylases or the regulatory subunit of trehalose biosynthesis, TslA, take over the function to produce trehalose, but no orthologs of these enzymes are found in *C. glabrata* (22, 68). These authors showed that *Af*Tps2 plays an essential role in cell wall integrity and fungal virulence (22). Whereas deletion of the *tsla* gene results in lower trehalose levels, the underlying mechanism is not clear as TslA lacks the catalytic site. However, the authors showed that TslA is regulating the activity and subcellular localization of a chitin synthase, providing a link between chitin synthase and trehalose metabolism (68).

The conclusions collectively suggest the involvement of trehalose in regulating both the resistance and tolerance to fluconazole, likely through its effects on membrane stability (23). This stabilization can influence the function of drug efflux pumps and associated transporters, leading to alterations in the sterol profile of the cell membrane. Elevated trehalose levels contribute to the stabilization of lipid bilayers, potentially enhancing the activity of membrane proteins, including those involved in drug efflux. Such stabilization may help mitigate the detrimental effects of fluconazole, allowing cells to better endure stress conditions. Moreover, trehalose’s role in modulating the sterol composition of the membrane can further impact drug permeability and efflux activity. By altering the membrane’s sterol profile, trehalose can influence the fluidity and functionality of membrane transporters, thereby affecting the efflux of toxic compounds, including antifungal agents. Our work again emphasized the potential of trehalose biosynthesis as a promising antifungal drug target (11, 17, 69). Despite several initiatives, a clear inhibitor of Tps1 or Tps2 is not under clinical investigation, as far as we know. One publication shows an inhibitory effect of N-(phenylthio) phthalimide (NPP) on the Tps2 enzyme of the plant fungal pathogen *Fusarium graminearum* (70) while another study used T6P as a lead compound to develop Tps1 inhibitors (35). Further research into developing Tps1 or Tps2 inhibitors is necessary to develop a novel class of antifungal drugs that could work alone or in combination with existing drugs.

## MATERIALS AND METHODS

### Yeast strains, plasmids, primers and media

All strains, plasmids, and primers used in this study are listed in Table S1 in the supplemental material. Cells were grown at 37°C in SC or YP medium supplemented with 100 mM glucose unless stated otherwise. YPD medium (1%, wt/vol, yeast extract, 2%, wt/vol, bacteriological peptone, 2% wt/vol glucose), YPG medium (3%, vol/vol, glycerol instead of 2%, wt/vol, glucose), and SC medium [0.079%, wt/vol, complete CSM (MP biomedicals, Santa Ana, CA, USA), 0.17%, wt/vol, yeast nitrogen base without amino acids or ammonium sulfate ((NH_4_)_2_SO_4_; Difco), and 0.5%, wt/vol, (NH_4_)_2_SO_4_, pH 5.5 (liquid) or pH 6.5 (solid)]. SC medium is supplemented with 5 mM glucose, 100 mM glucose, or glycerol (3%, vol/vol). RPMI 1640 with L-glutamine (Sigma-Aldrich, Saint Louis, MO, USA) is buffered with 0.165M morpholine propane sulfonic acid (MOPS) to pH 7. For solid media, 2% (wt/vol) agar (Difco) was added.

### Construction of *C. glabrata* mutant strains

The trehalose metabolism deletion strains were constructed in the *ATCC2001* background (71). The WT strain was transformed with the deletion cassette [the nourseothricin (NAT) marker flanked by FRT sites and a 100 bp region flanking the target gene] by making use of electroporation according to reference 14. The deletion cassettes were amplified from the pYC44 plasmid with promoter and terminator sequences of the gene of interest (*TPS1*, *TPS2,* and *NTH1*). After transformation, cells were plated on YPG agar medium supplemented with 200 µg/mL nourseothricin. Transformants were checked for insertion of the deletion cassette by PCR, and the correct strains were subsequently transformed with the pLS10 plasmid expressing the flippase enzyme to remove the NAT marker (300 µg/mL hygromycin selection). Removal of the NAT cassettes of the transformants was checked via PCR. Finally, the pLS10 plasmid was lost by growth on a nonselective YPG medium and checked by replating on YPG supplemented with 300 µg/mL hygromycin.

The complete coding sequence (CDS) of *ChTPSP* was amplified from the pSal4-ChTPSP plasmid (27). To express the bifunctional TPS–TPP enzyme, we followed the method outlined in reference 72, employing expression vectors designed for C-terminal fusions with mCherry fluorescent proteins (pYC56). Subsequently, we substituted the mCherry gene within the pYC56 vector with the *ChTPSP* gene, placing it under the control of a *TEF1* promoter and terminator. To facilitate the expression of the *ChTPSP* gene by replacing the *HIS3* gene, we constructed a replacement cassette by incorporating promoter and terminator sequences derived from the *C. glabrata HIS3* gene into the plasmid capable of expressing the *ChTPSP* gene. Subsequently, we generated a cassette containing the *ChTPSP* gene, a nourseothricin (NAT) marker flanked by FRT sites, flanked by the *HIS3* promoter and terminator sequences, thus forming the novel replacement cassette. Transformation of the WT, *tps1Δ*, and *tps2Δ* strains through electroporation was performed as previously described.

### Growth assay

The growth of the mutant strains in both liquid and solid media was monitored over time. For the liquid assay, growth was evaluated by spectrophotometric observation (OD_600_) over time using a Multiskan GO automated plate reader (Thermo Fisher, Waltham, MA, USA). The cultures were diluted in SC medium with glucose (5 mM or 100 mM), and growth was monitored for 36 h at 37°C. Growth curves were plotted as the average of three biological replicates (= independent mutants). For growth assays on solid medium, a 10-fold dilution series of the cultures was spotted on SC plates containing glucose (5 mM or 100 mM). The OD_600_ of the lowest dilution was 0.0001.

### Heat shock, oxidative, salt, membrane, and cell wall stress Treatments

The survival of *C. glabrata* cells was determined under various stress conditions using spot assays. Overnight cultures were washed three times with 1 × PBS and diluted to OD_600_ of 0.1 in 1 × PBS. Next, a 10-fold dilution series was made, and 5 µL of cells was spotted on SC plates with 2% (wt/vol) glucose. These plates were incubated for 48 h at 37°C, 39°C, or 42°C for the evaluation of heat stress. Oxidative and salt stress was evaluated on agar containing 6 mM H_2_O_2_ (Sigma-Aldrich) and agar containing 1.5 M NaCl, respectively. Membrane and cell wall stress was evaluated on agar containing 0.005% SDS and agar containing 2 mM CFW, respectively. Controls were maintained at 37°C without any treatment.

### Intracellular trehalose levels determination

Exponentially grown cells (25–50 mg) were harvested by centrifugation at 3,000 rpm (4°C) for 5 min. The culture medium supernatant was discarded, and the cells were resuspended in 1 mL ice cold Milli-Q water. The cells were broken by vigorously vortex in the presence of glass beads (0.75–1 mm) by means of the FastPrep (20 s, 6 m/s) (MP biomedical). Then, immediately transferred to a 95°C water bath for 5 minutes. Afterward, the sample was centrifuged at 12,000 rpm (4°C) for 5 min and 200 µL of the supernatant was used for analysis by the Shimadzu HPLC system using an Agilent 87 H column at 0.7 mL/min and a RID-20A detector (Shimadzu). High-quality water was used as eluent at a constant flow of 0.6 mL/min at 80°C. Pure trehalose (Sigma) was used as a standard.

### Intracellular Trehalose-6-P levels determination

Exponentially grown cells (10–20 mg) harvested by centrifugation at 3,000 rpm (4°C) for 5 min. The cells were extracted as described in Lunn et al. (73). T6P was assayed in extracts by anion-exchange HPLC using a Dionex HPLC system (Sunnyvale, CA, USA), coupled to an AB Sciex Q-Trap 6500 triple quadrupole mass spectrometer (as described in reference 59 with modifications as described in reference 74).

### RNA extraction and gene expression analysis by qRT-PCR

Cells were grown in SC medium plus 100 mM glucose for 4–5 h until exponential phase at 37°C, while shaking (200 rpm), and fluconazole was added to the cultures to give the final concentrations indicated, and the cells incubated for 60 min. The cells were centrifuged at 14,000 rpm (4°C) for 10 min and washed with 1 mL ice-cold water, resuspended in 1 mL TRIzol (ThermoFisher) and broken using glass beads (0.75–1 mm) in FastPrep machine (20 s, 6 m/s). RNA was extracted by respective addition of 360 µL chloroform and 350 µL isopropanol after which three washes with ethanol (70%, vol/vol) were conducted. RNA was treated with DNase enzyme (New England Biolabs) and converted to cDNA using the iScript cDNA synthesis kit (iScript cDNA synthesis kit; Bio-Rad). Real-time quantitative PCR (qPCR) was conducted using GoTaq polymerase (Promega) and the StepOnePlus real-time PCR device (ThermoFisher). *GAPDH* and *UBC13* were used as reference genes. Data were subsequently analyzed using qBasePlus software (75).

### Etest assays

Fluconazole MICs (minimum inhibitory concentration) were determined by using Etest strips (BioMérieux) on RPMI (2%, wt/vol, glucose) plates. Overnight cultures were washed twice with 1 × PBS, adjusted to OD_600_ 0.15 in 1 mL 1 × PBS, and restreaked on RPMI (2%, wt/vol, glucose) plates. Next, a fluconazole-containing gradient strip was placed in the middle of the plate. These plates were incubated at 37°C for 24–72 h. Subsequently, based on the size of the observed halo, a preliminary assessment of fluconazole tolerance was conducted, and the point of contact between the halo and the strip provided the means to evaluate the MIC_50_ value.

### Broth microdilution assays

The broth microdilution assays were based on the Clinical and Laboratory Standards Institute (CLSI) guidelines (76). Briefly, cells were harvested by centrifugation at 7,500 rpm (25°C) for 1 min from an overnight RPMI (2%, wt/vol, glucose) culture, washed three times with 1 × PBS, and diluted to around 2,500 CFU/mL (verified by plating serial dilution on YPD plates) in RPMI medium. Then, round-bottom, UV-sterilized 96-well microtiter plates were used, where all wells were filled with 100 µL diluted cells, 0–32 µg/mL fluconazole in 1/2 dilutions, and 80 µL RPMI medium. The plates were incubated at 37°C for 48 h. The MIC_50_ values were defined as the lowest concentration of the fluconazole that caused ≥50% decrease in optical density at OD_600_. To evaluate the drug tolerance of strains, we generated dose-response curves based on CFU counts for the WT, *tps2∆*, and *nth1∆* strains after incubation with fluconazole for 48 h. The *tps1∆* mutant was not included as this strain does not grow on glucose. The wells of the broth microdilution assay plate were resuspended, and each culture was diluted and plated on YPD plates. All experiments were conducted with three biological repeats.

### Sterol measurement

Sterols were extracted and analyzed based on Morio et al. (77) with some modifications. Cells were grown in SC medium plus 100 mM glucose for 3–4 h until exponential phase at 37°C, while shaking (200 rpm), and 20 µg/mL fluconazole was added to the cultures and incubated for 120 min. Cells were harvested by centrifugation at 3,000 rpm (4°C) for 5 min, washed twice with MilliQ H_2_O, and a pellet of 20 mg of cells was stored at −80°C. The pellet was resuspended (vortexing for 1 min) in 300 µL saponification medium (12.5 g KOH in 18 mL MilliQ H_2_O diluted to 50 mL with 98% ethanol), transferred to a capped glass vial, and incubated for 1 h in a shaking water bath at 80°C. Sterols were extracted by adding 100 µL MilliQ H_2_O and 400 µL hexane, including 1 µL of 5 mg/mL 5-a-cholestane as internal standard (Sigma, 47124), followed by vortexing for 3 min, 20 min phase separation and collection of 350 µL of the top (hexane) layer. A second extraction fraction was collected by adding 600 µL hexane, vortexing for 3 min, 20 min phase separation and collection of 550 µL of the top (hexane) layer. The two collected hexane fractions were combined and dried using vacuum centrifugation (Automatic Environmental SpeedVac System AES2010) for 30 min at room temperature. Sterol extracts were re-dissolved in 60 µL hexane and derivatized by adding 10 µL of silylating mixture (Sigma, 85432), short vortexing, and incubation at room temperature for 1 h. Derivatized extracts were shortly centrifuged to precipitate potential debris, and 50 µL of the extract was transferred to a smaller insert glass tube for GCMS analysis. The samples were analyzed using a Thermo Scientific gas chromatography-mass spectrometer [Trace 1300—ISQ QD equipped with a TriPlus RSH autosampler and a Restek Rxi-5ms capillary GC column (30 m × 0.25mmID)]. Helium was used as carrier gas with a flow rate of 1.4 mL/min. Injection was carried out at 250°C in split mode after 1 min and with a ratio of 1:10. The temperature was first held at 50°C for 1 min and then allowed to rise to 260°C at a rate of 50 °C/min, followed by a second ramp of 2 °C/min until 325°C was reached, that temperature was maintained for 3 min. The mass detector was operated in scan mode (50 to 600 atomic mass units), using electron impact ionization (70 eV). The temperatures of the MS transfer line and detector were 325°C and 250°C, respectively. Sterols were identified by their retention time relative to the internal standard (cholestane) and specific mass spectrometric patterns using Chromeleon software (version 7). Abundance was calculated relative to the internal standard, comparing the relative peak areas of the compounds. Sterol extraction and analysis of each strain were performed with three biological repeats.

### Rhodamine 6G (R6G) efflux analysis

R6G accumulation and glucose-induced efflux pump activity were assessed using a previously reported method with minor modifications (78, 79). WT and mutant cells were cultured in SC medium supplemented with 100 mM glucose for 4–5 h at 37°C with shaking at 200 rpm to reach the exponential growth phase. The cells were harvested by centrifugation at 5,000 × *g* for 5 min at 4°C, and the resulting pellets were washed twice with 25 mL of PBS. The cells were then resuspended in 1 mL of glucose-free PBS at a concentration of 1 × 10^8^ cells/mL and incubated at 37°C for 3 h to induce a starvation condition. Following this, the cells were resuspended in glucose-free PBS, and R6G was added to achieve a final concentration of 0.2 µg/mL. The cells were incubated for an additional 2 h at 37°C to allow for R6G uptake. The uptake process was halted by placing the culture on ice for 10 min. Cells were then separated by centrifugation at 12,000 × *g* and washed three times with glucose-free PBS to remove any extracellular R6G. R6G accumulation was quantified using a Cytek Guava flow cytometer equipped with a 525/30 emission filter, and the data were analyzed using the Python FlowCytometryTools package.

For the efflux activity assay, 2% glucose was added to the cells following the 2-h R6G accumulation to initiate energy-dependent R6G efflux. Samples were collected at 0, 2, 5, 10, 15, and 20 min, centrifuged at 9,000 × *g* for 2 min, and resuspended in PBS for further analysis. The fluorescence of the cells was quantified using a flow cytometer. Representative data from the analysis of 10,000 events are presented, with results expressed as the average fluorescence intensity of a population. Fungal cells not stained with R6G served as a negative control.

## SUPPLEMENTAL MATERIAL

Supplemental material aac.01349-24-s0001.pdf Figures S1 to S4, Table S1, and lists of primers and plasmids.

ASM does not own the copyrights to Supplemental Material that may be linked to, or accessed through, an article. The authors have granted ASM a non-exclusive, world-wide license to publish the Supplemental Material files. Please contact the corresponding author directly for reuse.
