# Supplementary material for: The stress-protectant molecule trehalose mediates fluconazole tolerance in Candida glabrata
Source: Antimicrob Agents Chemother. 2025 Jan 24;69(3):e01349-24. doi: 10.1128/aac.01349-24 (PMC11881567; doi:10.1128/aac.01349-24)
Supplement: Supplemental material — Figures S1 to S4, Table S1, and lists of primers and plasmids. [file aac.01349-24-s0001.pdf]

The stress-protectant molecule trehalose mediates fluconazole tolerance in *Candida glabrata*

Qingjuan Zhu[1], Stefanie Wijnants[1], Regina Feil[2], Wouter Van Genechten[1], Rudy Vergauwen[1], Odessa Van Goethem[1], John E. Lunn[2], Mieke Van Ende[1], Patrick Van Dijck[1,3,*]

[1]Laboratory of Molecular Cell Biology, Department of Biology, Institute of Botany and Microbiology, KU Leuven, Kasteelpark Arenberg 31, 3000-Leuven, Belgium

[2] Max Planck Institute of Molecular Plant Physiology, Am Mühlenberg 1, 14476 Potsdam-Golm, Germany

[3] Leuven One Health Institute, KU Leuven, Belgium

Supplementary material

Supplementary figures

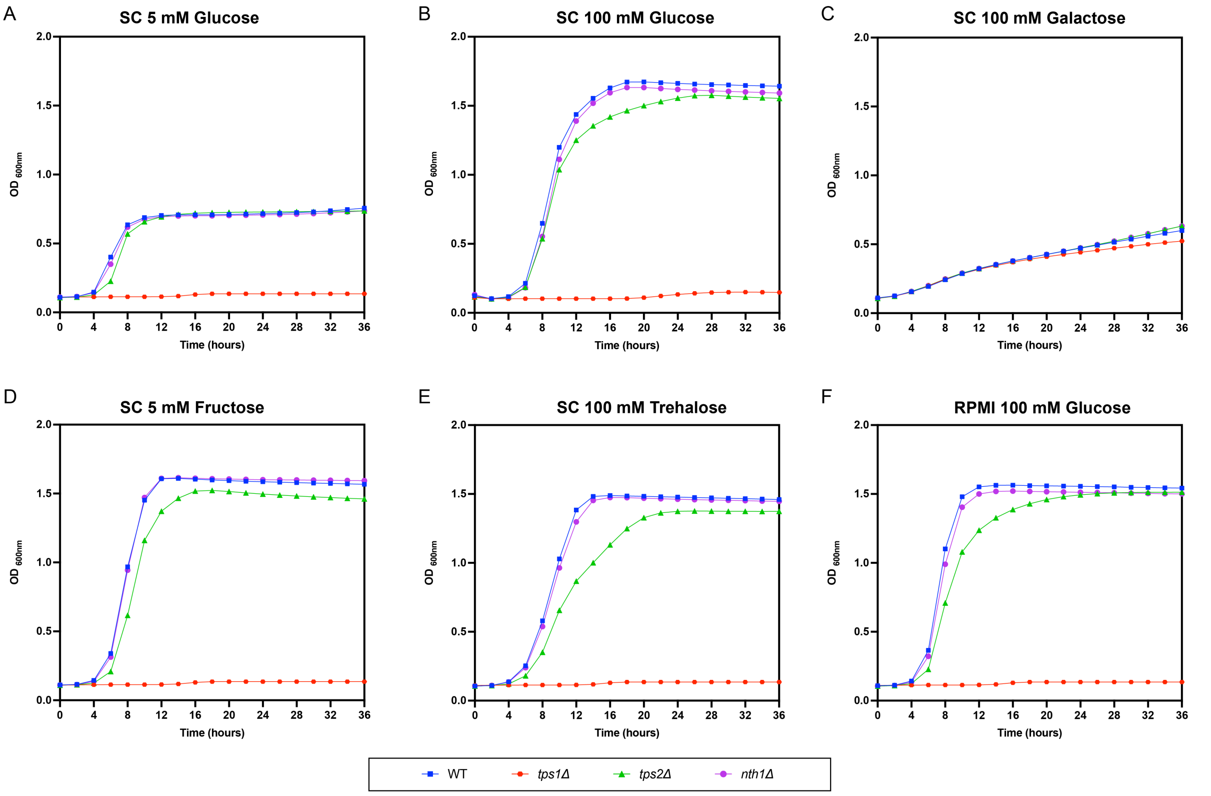

Fig S1. Deletion of *TPS1* gene affected utilization of glucose as a carbon source in liquid medium. WT, *tps1*Δ, *tps2*Δ and *nth1*Δ were grown at 37 °C in liquid medium using the Multiskan. The strains were grown in different media: SC supplemented with 5mM glucose (A), 100 mM glucose (B), 100mM galactose (C), 100 mM fructose (D), 100 mM trehalose (E) and RPMI with 100 mM glucose (F). The OD_{600} was followed over time for 48 h. The data represent the average of two independent experiments each consisting of three biological repeats and three technical repeats.

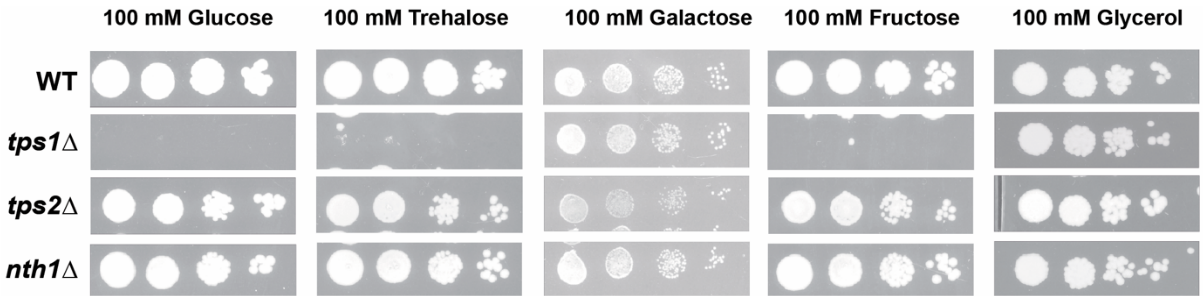

Fig S2. The *tps1*Δ strain showed a growth defect on solid medium with glucose, trehalose, and fructose as carbon source. The different strains were grown on solid SC medium supplemented with 100 mM glucose, 100mM trehalose, 100 mM galactose, 100mM fructose and 100 mM glycerol. The plates were incubated for 48 h at 37 °C before pictures were taken.

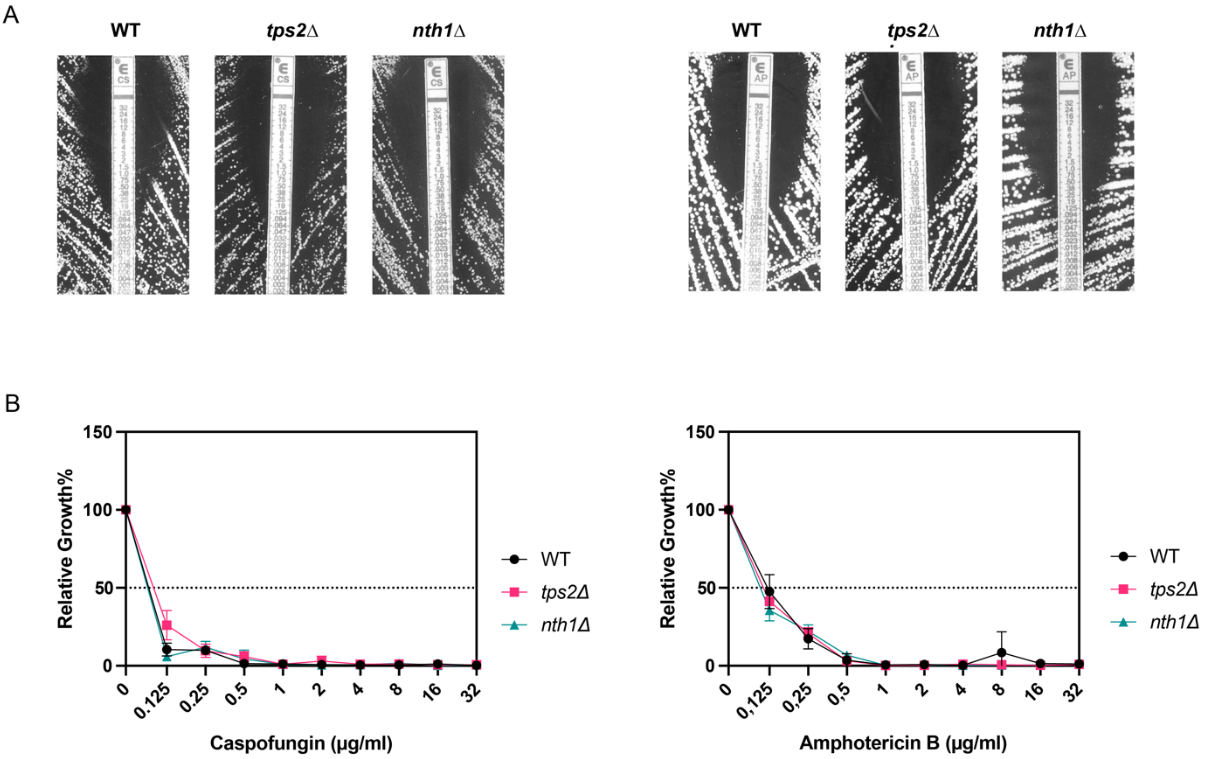

Fig S3 **Effect of *TPS1* and *TPS2* Gene Deletion on Resistance and Tolerance to Fluconazole in *C. glabrata*** (A) Etest analysis at 37°C (48 hours) showing a “halo” in regions of the medium with high concentrations of Caspofungin (left) and Amphotericin B (right) where the cells are unable to grow. (B) Growth profiles of mutant strains relative to the WT strain in a BDA in the presence of different concentrations of Caspofungin (left) and Amphotericin B (right).

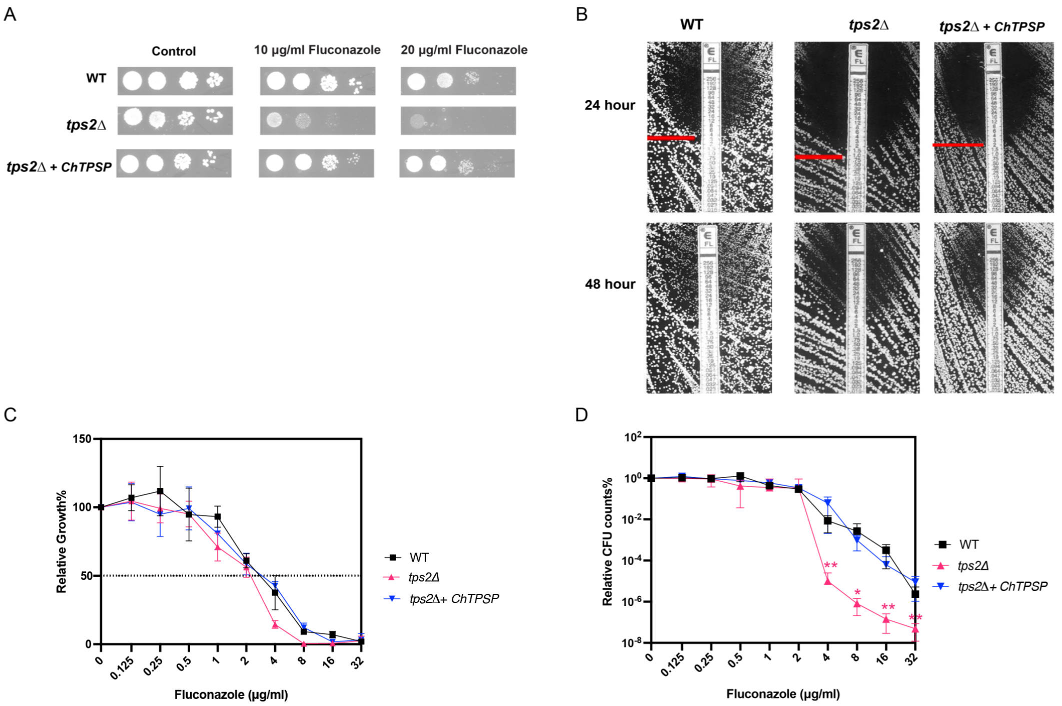

Fig S4. **Insertion of *ChTPSP* into the *tps2*Δ strain can restore resistance and tolerance to fluconazole in *C. glabrata*** (A) Serial dilutions of the WT, *tps2*Δ and *tps2*Δ+*ChTPSPS* strains spotted on SC glucose plates. Pictures were taken after 48 h of incubation at 37°C. (B) Etest analysis at 37°C (24 and 48 hours) showing a “halo” in regions of the medium with high concentrations of fluconazole where the cells are unable to grow. (C) Growth profiles of mutant strains relative to the WT strain in a broth dilution assay (BDA) in the presence of different concentrations of fluconazole. (D) Tolerance assay. Data reflect the relative percentage of colony forming units from the WT and mutant strains after 48 hours of incubation at 37°C. The experiment was conducted two times with three biological and three technical repeats, and representative results are shown.

Supplementary Tables

Supplementary Table 1 – Strains, plasmids and primers used in this study

List of strains:

**Species**	**Strain**	**Genotype**	**Source**
C. glabrata	WT	ATCC2001	[1]
C. glabrata	*tps1Δ*	*tps1*Δ:::FRT	This work
C. glabrata	*tps2Δ*	*tps2*Δ:::FRT	This work
C. glabrata	*nth1Δ*	*nth1*Δ:::FRT	This work

List of plasmids:

Plasmid	Description	Used for	Source
pYC44	NatMX cassette flanked by FRT sites	Construction of deletion cassette	[2]
pLS10	Flippase hph	Removal of resistance marker between FRT sites	[3]
pYC44-Ptps1-Ttps1	NatMX cassette with TPS1 promoter and termintor	Construction of deletion cassette	This work
pYC44-Ptps2-Ttps2	NatMX cassette with TPS2 promoter and termintor	Construction of deletion cassette	This work
pYC44-Pnth1-Tnth1	NatMX cassette with NTH1 promoter and termintor	Construction of deletion cassette	This work

List of primers:

Primer	Name	Sequence	used for
D-4441	CgTPS2_term_Fw_pYC44_XhoI	agagaataggaacttcgtccTCTGACGAGCTTATGATTTATG	Construction deletion cassette *TPS2*
D-4442	CgTPS2_term_rev_pYC44_XhoI	agctggtaccgggccccccTCCGCCAACAATTTAAGATACAATTTTTATAATAAATATATC	Construction deletion cassette *TPS2*
D-4443	CgTPS2_prom_Fw_pYC44_BamHI_EciI	cggccgctctagaactagtgGGCGGAGCGATACTTTTGCATAGC	Construction deletion cassette *TPS2*
D-4444	CgTPS2_prom_rev_pYC44_BamHI_EciI	tagaaagtataggaacttcgCTTTTTTTCTTGAATTGCTGTG	Construction deletion cassette *TPS2*
D-4435	CgTPS1_term_Fw_pYC44_XhoI	agagaataggaacttcgtccTCCAAAACAGATATGAACAAAG	Construction deletion cassette *TPS1*
D-4436	CgTPS1_term_rev_pYC44_XhoI	agctggtaccgggccccccTCCGCCCGGTATATCTCGATATATAAGAAC	Construction deletion cassette *TPS1*
D-4437	CgTPS1_prom_Fw_pYC44_BamHI_EciI	cggccgctctagaactagtgGGCGGAGTGATATAATCACCTGACC	Construction deletion cassette *TPS1*
D-4438	CgTPS1_prom_rev_pYC44_BamHI_EciI	tagaaagtataggaacttcgTGTCCTTTGTTATATTGCAG	Construction deletion cassette *TPS1*
9065	NatMX_Fw	catcatctgcccagatgcgaag	checking insertion pYC vectors
D-7295	CgTPS1-deletion_check Rev	CTGATGTGAATGGTACG	Checking transformants *tps1Δ*
D-7294	CgTPS1-deletion_check Fw	ATCCGACTTTGTTCTTCC	Checking transformants *tps1Δ*

D-4445	CgTPS2-deletion_check Rev	TGGGTTTAGTTTTAAGCCAC	Checking transformants *tps2Δ*
D-4446	CgTPS2-deletion_check Fw	ACAATAGATACCATCCCCTC	Checking transformants *tps2Δ*
D-6515	CgNTH1_prom_Fw_pYC44_BamHI_EciI	CGGCCGCTCTAGAACTAGTGggcggaAAAAAAAAAGGATCCTCCC	Construction deletion cassette *NTH1*
D-6514	CgNTH1_prom_rev_pYC44_BamHI_EciI	AGAGAATAGGAACTTCGTCCGTATAGAAAGTATAGGAACTTCGTATTGCACTATTTTTTTGGTGCTTTTTTGAAATGTTGCAC	Construction deletion cassette *NTH1*
D-6517	CgNTH1_term_rev_pYC44_XhoI	AGCTGGTACCGGGCCCCCCCGGCGGAGATCAAAGAAAAGTATAAATAATTAATTTAAAAATTAAAATAAATATATTTAATTTG	Construction deletion cassette *NTH1*
D-6516	CgNTH1_term_Fw_pYC44_XhoI	AGAGAATAGGAACTTCGTCCATTGAGACTCTTTATCTTTATATTCC	Construction deletion cassette *NTH1*
D-7292	CgNTH1-deletion_check Fw	GTTGATTGCTTTGACTCC	Checking transformants *nth1Δ*
D-7293	CgNTH1-deletion_check Rev	AAATAATGTGGTCCGAAC	Checking transformants *nth1Δ*
B-8458	GAPDH_qPCR_Fw	AACCGCTTCCGGTAACATCA	Reference gene
B-8459	GAPDH_qPCR_Rev	TGAAAGCCATACCGGTCAACT	Reference gene
B-7060	UBC13_Fw	TGCCCGAGGACTACCCTATG	Reference gene
B-7061	UBC13_Rev	AGCACGTCCAGGCAGATACG	Reference gene

B-4319	ERG11_qPCR_Fw	TGGTCAACATACTTCCGCTG	*ERG11* gene expression
B-4320	ERG11_qPCR_Rev	TTGGTACTCGACACCGTTGG	*ERG11* gene expression
D-7889	ERG25_qPCR_Fw	CGTCGGTATGCCAATCCTAT	*ERG25* gene expression
D-7890	ERG25_qPCR_Rev	GGAAGTCGTAACCGGAGTGA	*ERG25* gene expression
D-6103	ERG3_qPCR_Fw	TTATGATCCACGACGGTCAA	*ERG3* gene expression
D-6104	ERG3_qPCR_Rev	GTCCCACAAGGTGGTGAACT	*ERG3* gene expression
D-8039	ERG6_qPCR_Fw	GCTGACGAGGATGACGAAAT	*ERG6* gene expression
D-8040	ERG6_qPCR_Rev	CTGGCTTCTTAGCGACGAATAG	*ERG6* gene expression
D-8234	ERG26_qPCR_Fw	CCTCGTCTGCTGGTGTAATC	*ERG26* gene expression
D-8235	ERG26_qPCR_Rev	TCCATTGGGACTTCTGGTATTG	*ERG26* gene expression
D-8184	ERG27_qPCR_Fw	GACATCCAAGACCAGGAAAGTAG	*ERG27* gene expression
D-8185	ERG27_qPCR_Rev	GTCAGCAGACTGTCTTTCGATTA	*ERG27* gene expression
C-9193	PDR1_qPCR_Fw	CGGTGAGTTGGCCCTTACAA	*PDR1* gene expression
C-9194	PDR1_qPCR_Rev	TTTAATGTCGGCGGTTTCGC	*PDR1* gene expression
D-8324	UPC2A_qPCR_Fw	GGCTAACTCCACAGCCAAAA	*UPC2A* gene expression
D-8325	UPC2A__qPCR_Rev	GCACCTGGAGATGAAACC	*UPC2A* gene expression
D-8372	CDR1_qPCR_Fw	GCTTTCTACTGGTGTTGCTAATG	*CDR1* gene expression

D-8373	CDR1_qPCR_Rev	GCTTTCTACTGGTGTTGCTAATG	*CDR1* gene expression
B-7066	CDR2_qPCR_Fw	GTGCTTTATGAAGGCTACCAGATT	*CDR2 gene* expression
B-7067	CDR2_qPCR_Rev	TCTTAGGACAGAAGTAACCCATCT	*CDR2* gene expression

References:

1. Schwarzmüller, T., et al., *Systematic phenotyping of a large-scale Candida glabrata deletion collection reveals novel antifungal tolerance genes.* PLoS Pathog, 2014. **10**(6): p. e1004211.
2. Yáñez-Carrillo, P., et al., *Expression vectors for C-terminal fusions with fluorescent proteins and epitope tags in Candida glabrata.* Fungal Genetics and Biology, 2015. **80**: p. 43-52.
3. Van Ende, M., et al., *The involvement of the Candida glabrata trehalase enzymes in stress resistance and gut colonization.* Virulence, 2021. **12**(1): p. 329-345.
